# Juvenile overlap myositis: retrospective study about 20 cases

**DOI:** 10.1186/1546-0096-12-S1-P92

**Published:** 2014-09-17

**Authors:** Vanessa Remy Piccolo, Karen Lambot, Sylvain Breton, Marie Ferneiny, Cyril Gitiaux, Pierre Quartier, Christine Bodemer, Brigitte Bader-Meunier

**Affiliations:** 1 Paediatric Rheumatology, Hopital Necker, Paris, France; 2 Paediatric Radiology, Hopital Necker, Paris, France; 3 Dermatology and Paediatric Dermatology, Hopital Necker, Paris, France; 4 Paediatric Neurology, Hopital Necker, Paris, France

## Introduction

Inflammatory myopathies during childhood are clinically, biologically and pathologically heterogeneous.

## Objectives

The objective of this study is to collect cases of pediatric-onset overlap myositis to improve description and classification.

## Methods

Retrospective study of patients followed in Necker-Enfants Malades Hospital (Pediatric Rheumatology, Pediatric Dermatology) from january 2002 to march 2014, with overlap myositis defined by the association of inflammatory myositis and clinic features and/or biological signs of other connective tissue diseases (clinical features among polyarthritis, Raynaud syndrome, sclerodactyly, morphea, lupus skin rash, esophageal dyskinesia, pulmonary, intestinal, renal involvement and/or presence of overlap autoantibodies). For every patient we collected at diagnosis clinical features, biological profile, histological and radiological (MRI) data, treatments and evolution.

## Results

Twenty patients were included, 3 boys and 17 girls. The median age was 9 years 5 months (age between 3 years 9 months and 14 years 1 month). Every patient had myositis at diagnosis associated with polyarthritis (7 patients), Raynaud syndrome (4 patients), esophageal dyskinesia (2 patients), lung damage (interstitial lung disease or DLCO<70%, 5 patients). Two patients had sclerodactyly, 5 had morphea. One patient had typical dermatomyositis at diagnosis and developed pulmonary involvement and cutaneous sclerosis during evolution. Serum creatine kinase level was elevated for thirteen patients at diagnosis. Eighteen patients had autoantibodies at diagnosis or during evolution (ANA=17/20, anti-RNP=6/19, anti-cardiolipin=6/14, anti-DNA=2/20, anti-Ku=2/12, anti-synthetase=1/15, anti Pm Scl=1/7). Muscular MRI was performed on 12 patients (myositis=5/12, subcutaneous involvement=7, bone edema=6). Muscular biopsy was done on 16 patients (inflammation=8, capillary loss=6, CMH I expression=14, perifascicular atrophy=6). The first-line treatment included corticosteroid therapy (18 patients), alone (11 patients) or associated with hydroxycholoroquine (8 patients), methotrexate (5 patients), intravenous immunoglobulins (2 patients), mycophenolate mofetil (2 patients), azathioprine (1 patient) and plasmatic exchange (1 patient). The median duration of follow up is 20 months (from 7 months to 5 years): no patient died. Ten patients are in complete remission on the muscular plan. Nevertheless a majority develops a connective tissue disease (mixed connective tissue disease=6, polyarthritis=2, lupus=1, scleroderma=1).

## Conclusion

This is the first study emphasizes juvenile overlap myositis. Juvenile overlap myositis are heterogeneous diseases and differ from adult myositis. A specific pediatric classification is warranted to improve characterization and to adapt treatments. MRI and histological datas appear to be relevant, nevertheless it is essential to evaluate their reliability prospectively so that we can use them to improve classification and determination of prognostic factors.

## Disclosure of interest

None declared.

